# Prevalence of Human Papillomavirus (HPV) Genotypes in Cervicovaginal Secretions of Human Immunodeficiency Virus (HIV) Positive Indian Women and Correlation With Clinico-Virological Parameters

**DOI:** 10.3389/frph.2021.695254

**Published:** 2021-09-13

**Authors:** Mahima Lall, Lalit Dar, Neerja Bhatla, Pankaj Kumar, Aashish Choudhary, Sandeep R. Mathur, Rajiv M. Gupta

**Affiliations:** ^1^Armed Forces Medical College, Pune, India; ^2^All India Institute of Medical Sciences, New Delhi, India

**Keywords:** HPV, HIV, cervical cancer, genotypes, clinicopathological correlation, cytology

## Abstract

**Introduction and Background:** Both human papillomavirus (HPV) and the human immunodeficiency virus (HIV) are sexually transmitted. High-risk (HR) HPV types are a causal factor in cervical cancer. Persistent HPV infection in this subset of immunocompromised women results in faster disease progression. The study determined the prevalence of HPV genotypes in cervicovaginal secretions of HIV seropositive women and the correlation with CD4 counts and cytology.

**Method:** One hundred, non-pregnant, HIV-positive women of 18 years of age and above were enrolled in this cross-sectional study following approval by the institutional ethical committee. A written consent, questionnaire, followed by sample collection including a Papanicolaou (Pap) smear for cytology was undertaken. Cervicovaginal secretion samples were collected in the Digene^®^ specimen transport medium (STM) (Qiagen Gaithersburg Inc., MD, USA). HPV genotyping was carried out with PCR amplification of a 65-base pair (bp) fragment in the L1 region of the HPV genome using the short PCR fragment (SPF10) primers followed by reverse hybridization by line probe assay (LPA) using the INNOLiPA HPV Genotyping Extra kit (Fujirebio, Belgium). Quantitation of HPV-16 and−18 viral loads (VLs) was done by real-time PCR. Results of Pap smear cytology were correlated with CD4 counts and HPV-16 and−18 VLs.

**Results:** Mean age of the subjects was 34.9 years ± 7.2 years (median 33.0 years, range 24–60 years). HPV was detected in 62 of 93 (66.6%) samples. Twenty (32.25%) of these 62 samples harbored a single HPV genotype. Multiple genotypes (more than two) were detected in 38 (61.3%) samples. HPV-16 was the commonest genotype detected in 26 (27.9%) of all samples and 41.9% of HPV positive samples. Pap smear cytology was reported for 93 women included in the study. Women who had normal cytology were reported as negative for intraepithelial malignancy or lesion (NILM; *n* = 62; 71.36%), two women had a high-grade squamous intraepithelial lesion (HSIL), low-grade squamous intraepithelial lesion (LSIL; *n* = 11), atypical squamous cells of undetermined significance (ASCUS; *n* = 12). Those smears with inadequate material were reported as scant (*n* = 6). The median CD4 count was 363/cu.mm (range 39–787) in HPV-positive women compared to 423/cu.mm (range 141–996) in those HPV-negative women. Quantitation of HPV-16 and−18 VL was done in duplicate for samples positive by PCR reverse hybridization (INNOLiPA). Of these 20 samples (65%), 12 samples were positive by real-time PCR. The normalized HPV-16 VL ranged between 18 and 240,000 copies/cell. The normalized HPV-18 VL in cervical samples ranged between ~24 and 60,000 copies/cell.

**Conclusion:** HIV-positive women may be infected with multiple genotypes other than HPV-16 and−18. This may have implications on the vaccines available currently which target few specific genotypes only. Studies are required to determine the predictive role of HR HPV genotypes, in significant copy numbers especially in HIV seropositive women. It would be clinically relevant if the HPV VLs, cervical cytology, and CD4 counts are considered into cervical cancer screening programs for triage and follow-up of these women.

## Introduction

To achieve the goal of eliminating cervical cancer by 2030, there is a need for a multipronged approach involving a commitment to screen, test, and treat reliably (1). A “Point of Care” test, which screens and triages, is the need of the hour to improve diagnostics for this highly preventable cancer. Diagnostic accuracy of screening for cervical cancer also assumes greater importance in women living with the human immunodeficiency virus (WLHIV) (2). Human papillomavirus (HPV) is one of the commonest sexually transmitted infections (STIs) which has an important causal role in the pathogenesis of cervical cancer (3, 4). Worldwide cervical cancer ranks as the fourth most frequent and leading cause of death due to cancer in women, with an estimated 604,000 newly diagnosed cases and 342,000 deaths globally in 2020 (5, 6). In India, cancer of the breast followed by cancer of the oral cavity and cervix uteri are the leading sites involved among women (7). India also has a high burden of HIV/AIDS (8). Both HIV and HPV are sexually transmitted and are known to facilitate infection by each other (9, 10). Prevalence of HPV is higher in WLHIV (11, 12). This group of women is at a high risk of persistent infection by HPV and a faster progression to invasive cervical lesions. Quantitating HPV, in cervicovaginal secretions of the genital tract and correlating the viral load (VL) with the immunopathological parameters, may be clinically predictive, giving an early indication of cytological abnormalities at the transformation zone of the cervix. However, currently, there are no defined VL cutoff values, and this needs to be standardized. Papanicolaou (Pap) smear cytology and the grade of the lesion along with the HPV genotypes and VL could triage women for a clinical outcome and workup strategy. The present study describes the prevalence of HPV genotypes in WLHIV and the correlation of high-risk types with abnormal cytology, immunological parameters, such as CD4 counts and HPV VLs.

## Methodology

### Study Design and Subjects

One hundred consecutive, non-pregnant, HIV-positive women >18 years of age and reporting to the antiretroviral therapy (ART) clinic, Department of Obstetrics and Gynaecology and Department of Microbiology, of the All India Institute of Medical Sciences (AIIMS), New Delhi, India, were enrolled in this cross-sectional study. This study was conducted for 4 years following approval by the ethical committee. The inclusion criteria for including women in the study were HIV-positive women, aged 18–59 years, and who were willing to participate in the study. They were included irrespective of their treatment and CD4 counts. CD4 counts were done in the lab using a fluorescence-activated cell sorter (FACS) count analyzer (Becton-Dickinson Biosciences, San Jose, CA, USA). Women were excluded from the study if they were pregnant, hysterectomized, and had undergone a procedure on the cervix, or they were diagnosed with neoplastic lesions of the cervix. Written consent was taken from each participant, and a questionnaire was filled by a face-to-face interview. Details of their education, socioeconomic status, treatment history, most recent CD4 counts were noted.

### Sample Collection, Transport, and Storage

Consent was obtained, and the sample was collected after explaining the procedure to the women. Universal precautions were followed during the collection of cervicovaginal secretions. Cervicovaginal samples were collected for conventional cytology for Pap stain followed by samples for HPV DNA genotyping. The Digene^®^ cervical brush was used for collecting cells from the endocervix, it was rotated 3–5 times under direct vision and placed in the Digene specimen collection tube, containing 1 ml of the specimen transport medium (STM, Qiagen Gaithersburg Inc., USA). Pap smear slides for cytology were put in a fixative and sent to the cytopathologist for reporting. Doubtful or undetermined smears were reviewed independently by two cytopathologists. The cytopathologists were blinded to the HPV molecular assay results. The collection tubes with the cervical samples for HPV DNA testing were transported on ice to the laboratory and stored at −70°C until further processing.

### Sample Processing for HPV Genotyping

The specimen in the STM was centrifuged and aliquoted for HPV DNA extraction. Detection and genotyping of HPV were done using the INNOLiPA HPV Genotyping test kit (Fujirebio, Belgium), a line probe assay (LPA) based on the principle of reverse hybridization (13, 14). This is a highly sensitive assay with an amplification step that has been included before hybridization, which detects a 65 base pair (bp) region of the L1 gene (15). The QIAamp Viral DNA Blood Mini Kit (QIAGEN, Hamburg, Germany) was used for DNA extraction from the cervical secretions as per the instructions of manufacturers. DNA was extracted from 200 μl of the divided STM sample aliquot and eluted in 100 μl of elution buffer. Negative and positive controls were included in each DNA extraction run for quality control. The eluted DNA was stored at −80°C.

#### HPV Genotyping by LPA

Human papillomavirus genotyping was carried out by INNOLiPA HPV Genotyping Extra kit, (Fujirebio, Belgium). The kit detects 28 HPV types, such as low-risk (LR) and high-risk (HR) HPV types—HPV-6LR, −11LR,−16HR, −18HR,−26pHR, −31HR, −33HR, −35HR, −39HR, −40LR, −43LR, −44LR, −45HR, −51HR, −52HR, −53pHR, −54, −56HR, −58HR, −59HR, −66PHR, −68HR, −70LR, −69/71, −73HR, and −82HR. The sequence variation within the SPF-10 primers allows the recognition of these different HPV genotypes (16). An additional primer pair for the amplification of the human leukocyte antigen HLA-DPB1 cellular gene is incorporated to monitor sample quality. The HPV DNA amplification was done using the short PCR fragment (SPF10) primer set, targeting the L1 gene followed by reverse hybridization. PCR amplification was done using 10 μl of the extracted DNA, 37.7 μl amplification mix (biotinylated SPF primers, dNTPs, and MgCl_2_), and 2.3 μl of enzyme mix (AmpliTaq Gold DNA polymerase) reagents provided in the INNOLiPA HPV Genotyping Extra assay (Fujirebio, Belgium). The PCR reaction was a 50 μl reaction performed in the thermal cycler as follows: decontamination at 37°C for 10 min, denaturation at 94°C for 9 min, 45 cycles of denaturation at 94°C for 30 s, annealing at 52°C for 45 s, and extension at 72°C for 45 s. The biotinylated amplified HPV DNA PCR products were denatured and hybridized to oligonucleotide probes immobilized on a nitrocellulose membrane. After washing thoroughly, streptavidin-conjugated alkaline phosphatase was added, which bound to any biotinylated hybrid formed. A colorimetric reaction gave a purple/brown precipitate indicating a positive band. A specific pattern of bands as specified by the interpretive chart provided by the manufacturers was used to find out the HPV type, and results were read visually. A sample was considered positive if one of the defined type-specific banding patterns along with one of the HPV control lines was positive.

#### Interpretation of Pap Smears

Papanicolaou smear reporting was as per the Bethesda system for reporting cervical cytology (17). A smear reported as negative for intraepithelial lesion or malignancy (NILM) meant that there were no cytological abnormalities in the cells from the cervix. Positive cytology was reported if there was an atypical squamous lesion of undetermined significance (ASCUS) or ASCUS-H, which included low-grade squamous intraepithelial lesion (LSIL) and high-grade squamous intraepithelial lesion (HSIL). A high-grade lesion was progressing over time and developing into invasive cervical cancer (ICC).

#### Quantitation of VLs for HPV-16 and −18

Quantification of HPV-16 and−18 VLs was done for samples that were positive for these by LPA using the TaqMan assay performed on the ABI Prism 7500 [Applied Biosciences (Thermofisher Scientific, Invitrogen USA)] and the Step One Plus platform. The targeted genome segments were the E6 and E7 regions of the HPV genome. Amplification was performed for HPV-16,−18, and one internal control (housekeeping gene) glyceraldehyde-3-phosphate dehydrogenase (GAPDH) using primers and probes designed in-house by using published references (18). The cloned plasmid containing the target genes with the known copy numbers was used to make the standard curve for the TaqMan absolute quantification assay. Standardization of TaqMan real-time PCR assay for quantitation of HPV-16 and−18 was done using the recombinant plasmid DNA to prepare in-house standards. The PCR positive controls for HPV-16, HPV-18, and GAPDH were obtained from viral stocks available in the laboratory. The E6 and E7 open reading frames (ORFs) from the HPV-16 and HPV-18 reference nucleotide sequences (GeneBank Accession Number K02718 and X05015 for HPV-16 and HPV-18, respectively) were searched for suitable primer and probe-targeted sites. The basic local alignment search tool (BLAST) was used to select nucleotides spanning 231–434 bp. Primers were designed using the Invitrogen Perfect Primer site which successfully amplified a 301 bp product of the E6 gene of HPV-16. For HPV-18 primer, target sites spanned nucleotides 667–803, and 251 bp product of the E7 gene was successfully amplified by the custom-designed primers and 538 bp product of GAPDH. These amplicons were successfully cloned into a TOPO vector using the TOPO TA cloning kit (Invitrogen, USA). *Escherichia coli* (*E. coli*) cells were transformed, and the presence of the insert was confirmed by the blue/white screening of the transformed colonies grown overnight on the Luria Bertani (LB) agar. The plasmid was extracted, and the concentration of the constructed cloned plasmid was measured by spectrophotometry. On measuring, the plasmid DNA concentration for HPV-16 was 50.0 ng/μl, HPV-18 plasmid DNA concentration was 187.0 ng/μl, and constructed cloned GAPDH plasmid DNA concentration was 140.0 ng/μl. The 10-fold serial dilutions of the constructed plasmid containing the target gene were prepared, from 10^7^ to 10^1^ copies/μl, which corresponded to the cycle threshold (Ct) range of 18.3–41.95 on real-time PCR. The Ct values of HPV-16,−18, and GAPDH standards measured in duplicate are provided in the Supplementary Material. The software determined the Ct value where the fluorescence intensity reaches the baseline. A standard curve was plotted between the Ct value and the log of the initial copy number of the standards for HPV-16, HPV-18, and GAPDH. The Ct value showed the expected inverse relation with the initial copy number of the standards, generating the graph as a straight line with a negative slope. The following conversion formula was used for calculating copy numbers from the plasmid DNA concentration for the calculation of the VL, which was converted to copies per cell.


$$\begin{array}{l} Calculation\;for\;copy\;number\;(in\;copies/\;\mu l)\\ =\;\underline{Plasmid\;DNA\;Concentration\;(g/\mu l)}\times \underline{Avogadro's\;Number}\\ \;Molecular\;weight\;(g/mol) \end{array}$$

The cloned plasmid containing the target gene (with known copy number) was used to make the standard curve for the TaqMan absolute quantification assay. Once the standard curves at different dilutions were prepared for HPV-16, HPV-18, and GAPDH in-house, and assay standardized, the samples were run along with these standards.

##### HPV-16 and HPV-18 Real-Time PCR: Primer-Probe and Reaction Conditions

Viral loads were detected by amplifying a 223 bp fragment of the E6 region of the HPV-16 genome and a 137 bp fragment of the E7 region for HPV-18. Real-time quantitative PCR was performed by adding 5 μl of extracted DNA to the TaqMan master mix, with 800 nM forward and reverse primers, and a 200 nM labeled probe, in a final volume of 25 μl. For HPV 16 PCR, the forward primer used was 5′ ATGACTTTGCTTTTCGGGAT 3′, reverse primer used was 5′CTTTGCTTTTCTTCAGGACA3′, and probe primer used was 5′ACGGTTTGTTGTATTGCTGTTCTA3′ (FAM-TAMRA). Thermal cycling conditions were as described previously (18). An initial 50°C for 2 min followed by pre-PCR denaturation step at 95°C for 10 min, and then 45 cycles of 95°C for 15 s, and 55°C for 1 min. For HPV-18 real-time PCR, the forward primer used was 5′ ATG TCA CGA GCA ATT AAG C 3′, reverse primer was 5′ TTC TGG CTT CAC ACT TAC AAC A 3′, and probe used was 5′CGG GCT GGT AAA TGT TGA TG 3′ (FAM-TAMRA). The results were expressed as HPV copies per cell.

##### GAPDH Real-Time PCR: Primer-Probe and Reaction Conditions

Cell's GAPDH DNA from each sample was tested in parallel, as an internal control, and for cell number estimation. The reaction mixture contained 2.5 μl of extracted DNA, 5 units of AmpliTaq Gold DNA polymerase, 4 mM MgCl_2_, 200 μM dNTPs, 0.2 μM of each primer, 0.1 μM probe, and 1 × PCR buffer. The sequence of GAPDH forward primer used was 5′ CTC CCC ACA CAC ATG CAC TTA-3′, the reverse primer used was 5′CCT AGT CCC AGG GCT TTG ATT-3′, and probe used was 5′-AAA AGA GCT AGG AAG GAC AGG CAA CTT GGC-3′ (VIC-TAMRA). The volume was made up to 25 μl. The thermal cycling conditions were as follows: an initial hold at 50°C for 2 min followed by pre-PCR denaturation step at 95°C for 12 min, and then 50 cycles of 95°C for 15 s, and 55°C for 30 s. The results were expressed as GAPDH copies per cell (19).

### Statistical Analysis

All the data were compiled and entered in a Microsoft Excel spreadsheet and analyzed using statistical product service solutions (SPSS) software v25 and STATA version 17 (Stata Corporation, College Station, TX, USA). Descriptive statistics, such as mean and range, were calculated for continuous variables. Frequency and percent values were computed for qualitative variables. A *p* < 0.05 was considered statistically significant.

## Results

One hundred HIV-1 positive women were enrolled; none of these women was HIV-2 positive. The mean age of the subjects was 34.9 years ± 7.2 years (median 33.0 years, range 24–60 years). The demographic details have been published elsewhere (20). Samples from seven women were found to be inadequate thus, 93 samples were included in the study.

### HPV Types Detected

Human papillomavirus DNA of any genotype was detected in 62 (66.6%) of the 93 samples, from 58 of which, the HPV detected could be typed, while four samples were untypeable (HPV-X). In all, 20 (32.25%) of these 62 samples harbored a single genotype, and multiple genotypes were detected in 38 (61.3 %) samples. HR HPV genotypes were present in 37 (39.8%) of the 93 samples tested, comprising 59.7% of the 62 HPV DNA positive samples by PCR-hybridization.

### Distribution of HPV-16 and HPV-18

Either HPV-16 and/or HPV-18 was present in 30 (32.3%) of 93 samples and 48.4% of the 62 HPV DNA positive samples. HPV-16 was detected in 26 (27.9%) of all samples and 41.9% of the HPV positive samples, while HPV-18 was detected in 5 (5.4%) of all samples and 8.1% of the HPV positive samples. One sample had both HPV-16 and HPV-18 on PCR hybridization.

### Frequencies of Different HPV Genotypes in the Patient Samples

**Figure 1:**
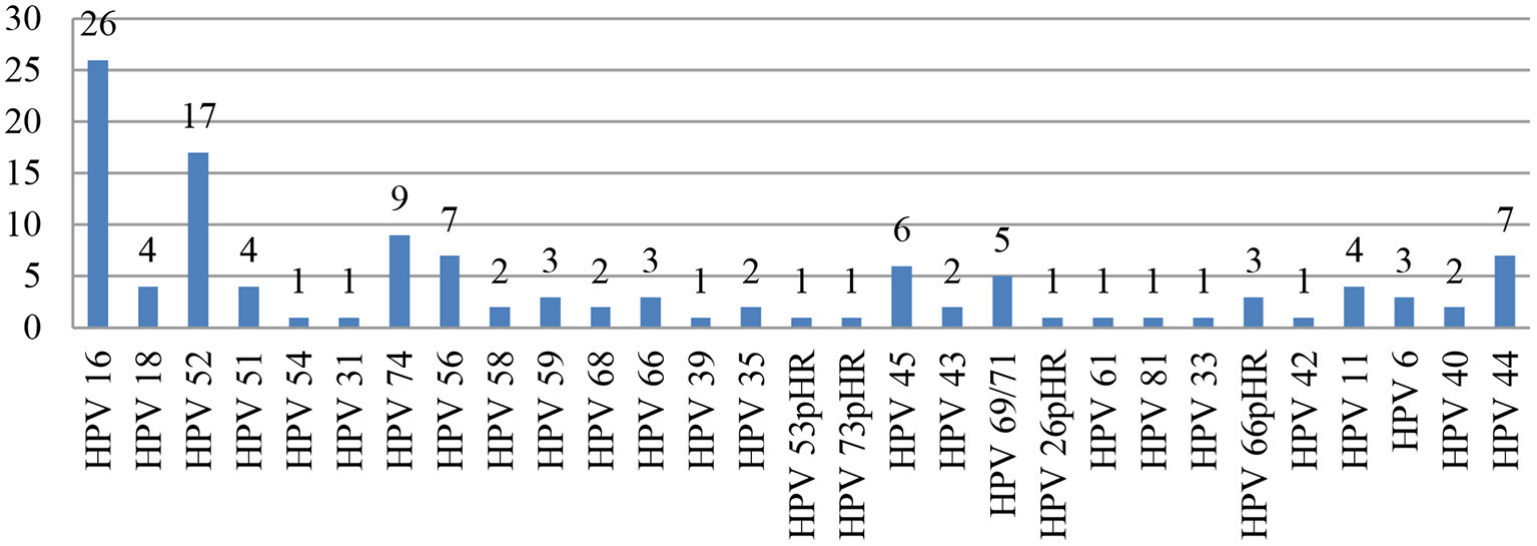
Frequencies of different HPV Genotypes in study samples.

The most common genotypes detected were HPV-16^*^ (*n* = 26) and HPV-52 (*n* = 17). The other genotypes detected in decreasing order were as follows: HPV-74, −56^*^, −44, −45^*^, −69/71, −18^*^, −51^*^, −11, −59^*^, −66pHR, −6, −58^*^, −68^*^, −35^*^, −43, −40, −54, −31^*^, −39^*^, −53pHR, −73pHR, −26pHR, −61, −81, −33^*^, and −42. (^*^High-risk HPV type; pHR: probable high-risk HPV type; Figure 1).

The patients who were positive for HPV on reverse hybridization were divided into four groups for analysis. The four groups were as follows: **Group I**, any HPV positive; **Group II**, high-risk (HR) HPV positive; **Group III**, HPV-16 positive; **Group IV**, HPV-16, and HPV-18 positive.

### Abnormal Cytology and HPV Genotypes

After excluding six samples of the 93 samples reported as scant by the cytopathologist, results for 87 samples were available. Sixty-two (71.36%) women had normal cytology.

**Figure 2:**
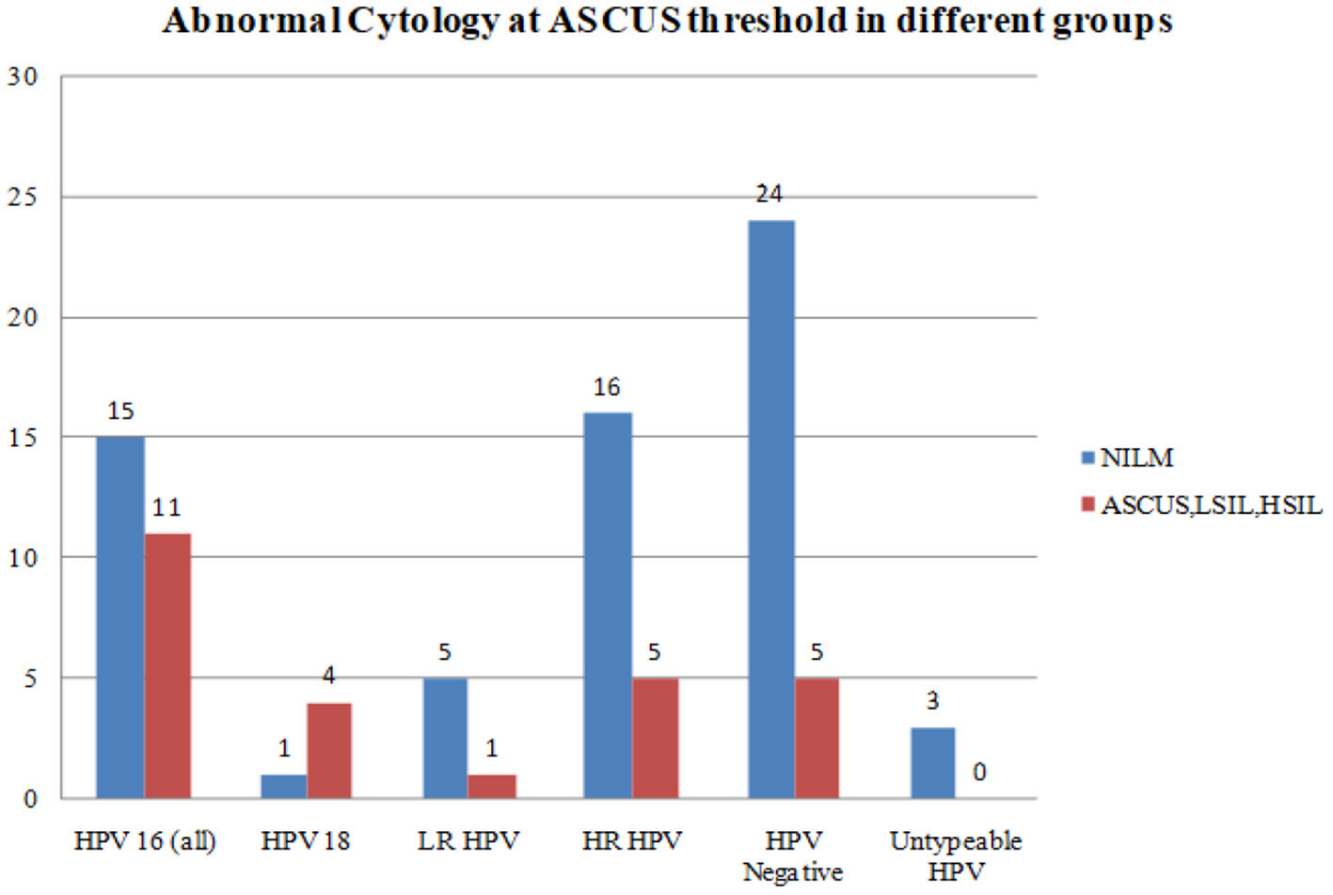
Abnormal cytology in different HPV genotypes at ASCUS threshold.

Abnormal cytology was reported in 25 women as HSIL (*n* = 2), LSIL (*n* = 11), and ASCUS (*n* = 12). HPV-16 and HPV-18 positive samples were associated with a higher degree of cytological abnormalities at ASCUS threshold as compared with LR HPV, all HR HPV taken together, or HPV negative samples (Figure 2).

### Clinicopathological Correlation of Pap Cytology and PCR Hybridization

In this study, while the percentage positivity was similar for HPV-16 in HIV-positive women with normal cytology (19.4%) and ASCUS (25%), in LSIL, the representation of HPV-16 was much higher (72.7%). There were only two HSIL cases in this study, one of which was positive for HPV-16, and the other had HPV-73 as the solitary HPV type. The association between Pap smear abnormality and HPV DNA positivity by PCR hybridization, in the four groups, was analyzed (Table 1). There was a significant difference between cytological abnormalities and PCR hybridization positivity across all groups, with *p*-values ranging from 0.001 to 0.043, which was most pronounced in the HPV negative group.

**Table 1:** Comparison of cytological grading with PCR hybridization.

**Group**	**Cytology**	**HPV by PCR hybridization**		***p*-value**
		Positive (*n* = 62)	Negative (*n* = 31)	
Group I (any HPV positive) (*n* = 93)	HSIL (*n* = 2)	2 (100.0%)	0 (0.0%)	0.091
	LSIL (*n* = 11)	11 (100.0%)	0 (0.0%)	
	ASCUS (*n* = 12)	8 (66.7%)	4 (33.3%)	
	NILM (*n* = 62)	37 (59.7%)	25 (40.3%)	
	Scant (*n* = 6)	4 (66.7%)	2 (33.3%)	
Group II—all (HRHPV positive, including HPV-16,−18) (*n* = 52) vs. LR and negative		Positive (*n* = 52)	Negative (*n* = 41)	
	HSIL (*n* = 2)	1 (50.0%)	1 (50.0%)	0.011
	LSIL (*n* = 11)	11 (100.0%)	0 (0.0%)	
	ASCUS (*n* = 12)	6 (50.0%)	6 (50.0%)	
	NILM (*n* = 62)	30 (48.4%)	32 (51.6%)	
	Scant (*n* = 6)	2 (50.0%)	4 (50.0%)	
Group III (HPV-16 positive) (*n* = 26)		Positive (*n* = 26)	Negative (*n* = 67)	
	HSIL (*n* = 2)	1 (50.0%)	1 (50.0%)	0.006
	LSIL (*n* = 11)	8 (72.7%)	3 (27.3%)	
	ASCUS (*n* = 12)	3 (25.0%)	9 (75.0%)	
	NILM (*n* = 62)	12 (19.4%)	50 (80.6%)	
	Scant (*n* = 6)	2 (33.33%)	4 (66.67%)	
Group IV (HPV-16,−18 positive) HPV-16 and−18 positive by PCR hybridization		Positive (*n* = 30)	Negative (*n* = 63)	
	HSIL (*n* = 2)	1 (50.0%)	1 (50.0%)	<0.001
	LSIL (*n* = 11)	10 (90.9%)	1 (9.1%)	
	ASCUS (*n* = 12)	4 (33.33%)	8 (66.67%)	
	NILM (*n* = 62)	13 (20.97%)	49 (79.03%)	
	Scant (*n* = 6)	2 (33.33%)	4 (66.67%)	

*LR, low risk; HSIL, high-grade squamous intraepithelial lesion; LSIL, low-grade squamous intraepithelial lesion; ASCUS, atypical squamous cells of undetermined significance; NILM, negative for intraepithelial malignancy or lesion*.

### HPV VL Estimation

Of the 26 cervical samples positive for HPV-16 by PCR reverse hybridization (INNOLiPA), real-time PCR was performed for 20 samples (in duplicate), of which the housekeeping gene GAPDH could not be amplified in two samples. The results of 18 samples are shown in Table 2. Of these, 13 (65%) samples were positive by real-time PCR. The normalized VL ranged between 10 and 240,000 copies/cell.

**Table 2:** HPV-16 viral load, cytology, and CD4 counts (*n* = 18).

**S.no**.	**Patient ID**	**HPV-16 copies/cell**	**Pap smear**	**CD4 (cells/mm^**3**^)**
1	Patient ID 9	0	LSIL	565
2	Patient ID 17	1,816	LSIL	420
3	Patient ID 21	10,854	LSIL	322
4	Patient ID 37	187	NILM	598
5	Patient ID 46	293	HSIL	187
6	Patient ID 47	743	LSIL	350
7	Patient ID 51	0	NILM	328
8	Patient ID 22	10	NILM	676
9	Patient ID 21	0	NILM	691
10	Patient ID 42	914	LSIL	520
11	Patient ID 45	36	ASCUS	173
12	Patient ID 94	1,400	LSIL	147
13	Patient ID 59	0	NILM	342
14	Patient ID 60	0	NILM	569
15	Patient ID 66	243,935	NILM	280
16	Patient ID 57	1,576	LSIL	432
17	Patient ID 86	3,145	LSIL	245
18	Patient ID 99	4	ASCUS	366

*HSIL, high-grade squamous intraepithelial lesion; LSIL, low-grade squamous intraepithelial lesion; ASCUS, atypical squamous cells of undetermined significance; NILM, negative for intraepithelial malignancy or lesion; 0-VL, not detected/below level of detection*.

The normalized HPV-18 VL in cervical samples was in a range of ~24–60,000 copies/ cell.

### Correlation of HPV With CD4 Counts and Clinicopathological Data

The CD4 count of the study group (*n* = 93) ranged from 39 to 1,056 cells/mm^3^ (mean, 414.6 cells/mm^3^ and median, 389 cells/mm^3^). Pap smear cytology results were correlated with the CD4 category. Distribution in different CD4 categories, as per the Centre for Disease Control (CDC) classification, along with the cytology in each category is shown in Tables 3, 4. The Pap positivity was higher (60.0%) in subjects with CD4 counts <200 cells/mm^3^ as compared to those with CD4 counts >200 (22.89%).

**Table 3:** Frequency of Pap positivity in CD4 categories (*n* = 87).

		**Pap cytology**		**Total**	**Percent (%)**	***p*-value**
		**Positive**	**Negative**			
**CD4 categories**	**0–199** (*n* = 10)	6	4	10	60.00	
	**200–499** (*n* = 54)	13	36	49	26.53	0.068
	**≥500** (*n* = 29)	6	22	28	21.42	
Total		25	62	87	28.73	

*Pap cytology positive—HSIL, high-grade squamous intraepithelial lesion; LSIL, low-grade squamous intraepithelial lesion; ASCUS, atypical squamous cells of undetermined significance; Pap cytology negative—NILM, negative for intraepithelial malignancy or lesion*.

*Bold values indicate self-explanatory*.

**Table 4:** Distribution of Pap categories in CD4 categories (*n* = 87).

**CD4 categories cells/mm^**3**^**	**NILM**	**ASCUS**	**LSIL**	**HSIL**
**0–199** (*n* = 10)	4 (40.0%)	3 (30.0%)	2 (20.0%)	1 (10.0%)
**200–499** (*n* = 54)	36 (66.7%)	6 (11.1%)	6 (11.1%)	1 (1.9%)
**≥500** (*n* = 29)	22 (75.9%)	3 (10.3%)	3 (10.3%)	0 (0.0%)

*Samples with scant material in cytology were excluded (n = 6)*.

*HSIL, high-grade squamous intraepithelial lesion; LSIL, low-grade squamous intraepithelial lesion; ASCUS, atypical squamous cells of undetermined significance; NILM, negative for intraepithelial malignancy or lesion*.

*Bold values indicate self-explanatory*.

It was observed that as the CD4 counts decreased, the proportion of abnormal cytology increased (ASCUS/LSIL/HSIL; Table 2).

### HPV Positivity, PCR-Hybridization, and CD4 Category

It was noted that the median CD4 count in the HPV-positive subset (*n* = 62) was 363 cells/mm^3^, range (39–1,056), while in the HPV-negative subset it was higher at 423 cells/mm^3^, range (141–883), though the difference was not significant. Infection with HPV types other than HPV-16 and HPV-18 was associated with the lowest median CD4 count (168 cells/mm^3^) range (138–890).

Distribution in different CD4 categories, as per the CDC classification, revealed HPV positivity was higher (9/10; 90%) in those with CD4 counts < 200 cells/mm^3^ than in those with CD4 counts >200 cells/mm^3^ (53/85; 63.9%), but the difference was not significant (*p* = 0.273) *p*-value < 0.05 statistically significant.

The logistic regression analysis was done, the odds ratio (OR) of having a positive hybridization in women with CD4 > 200 cells/mm^3^ was 0.264 times lower than those with CD4 < 200 cells/mm^3^. The 95% CI was 0.03–2.29 and was not significant.

Similarly, a Pap positivity had 0.360 odds of having a positive hybridization as compared with a negative Pap. The 95% CI was 0.108–1.19 and was not significant. A two-predictor logistic model was fitted to the data to test if CD4 and pap predicted the outcome. However, they were not found significantly related to the outcome. The patients who were positive for HPV on reverse hybridization were divided into four groups for analysis. Group I, any HPV positive (*p* = 0.273); Group II, high-risk (HR) HPV positive (*p* = 0.62); Group III, HPV-16 positive (*p* = 0.999); Group IV, HPV-16 and HPV-18 positive (*p* = 0.896).

Table 1 shows that cytological abnormalities were significantly higher with HPV PCR hybridization positivity for HRHPV types (*p* = 0.011), for HPV-16 (*p* = 0.006), and most significantly, for HPV-16 and/or 18 (*p* < 0.001; Groups II–IV), but not when the sample was positive for any HPV type (Group I).

The nonparametric Pearson's Chi-square test showed the association between the categorical variables that the Pap smear cytology was differently distributed in samples harboring HRHPV-16 and−18 when compared with the samples, which were negative for any cytological abnormality. The Chi-square value was 48.24, and the Likelihood ratio of 29.61 was significant.

### HPV-16 and HPV-18 VLs and Correlation With Cytology

**Figure 3:**
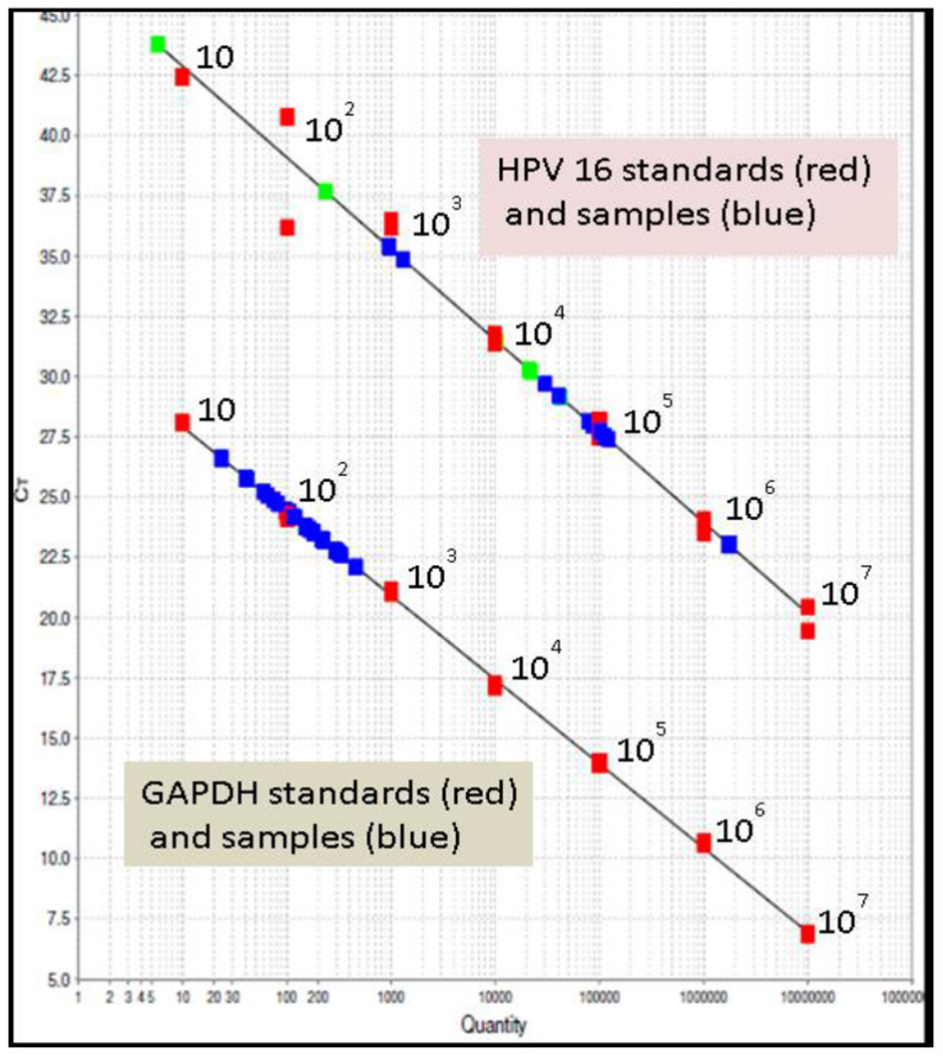
Standard curve for HPV-16 viral load quantitation; upper line in the graph shows HPV-16 standards (red), HPV-16 samples (blue). The lower line in the graph shows glyceraldehyde-3-phosphate dehydrogenase (GAPDH) standards (red) and samples (blue). All in duplicate. Seen clustering in the range of 10–10^3^ is sample GAPDH, a housekeeping gene for normalizing the results; shown in green are the known positive samples, run as controls.

The viral load of HRHPV-16 along with CD4 counts and cytology is shown in Table 2. VL for HRHPV-18 along with CD4 counts and cytology is shown in **Table 6**. The standard curve for HPV-16 VL quantitation is shown in Figure 3.

A statistically significant correlation was seen in HPV-16 VL and the presence of abnormal Pap cytology (Table 5).

**Table 5:** HPV-16 viral loads vs. Pap cytology.

**PAP cytology**	**HPV-16 viral load**		**Total**	***p*-value**
	**Detected**	**Not detected**		
Positive	10	1	11	0.047
Negative	3	4	7	
Total	13	5	18	

*p-value statistically significant*.

Human papillomavirus-18 real-time PCR was carried out in duplicate for five samples, which were positive by PCR reverse hybridization. Of these, three samples had detectable VL. Cytological abnormality was noted in two of these three samples, their Pap smears were reported as ASCUS-H (high grade) and LSIL. CD4 counts of the HPV-18 positive women were in the range of 112–533 cells/mm^3^ (Table 6).

**Table 6:** HPV-18 viral loads, cytology, and CD4 count.

	**Viral load (viral copies/cell)**	**Cytology**	**CD4 cells/mm^**3**^**
S1	4,195.07	**LSIL**	112
S2	33.90	NILM	432
S3	0	**ASCUS-H**	533
S5	0.5	**LSIL**	295

*LSIL, low-grade squamous intraepithelial lesion; ASCUS: atypical squamous cells of undetermined significance; ASCUS-H, atypical squamous cells of undetermined significance-high grade; NILM, negative for intraepithelial malignancy or lesion; 0, Not detected/below the limit of detection*.

*Bold values indicate self-explanatory*.

## Discussion

In this study in WLHIV, the prevalence of HPV DNA was 66.6% (*n* = 62) of the 93 samples. The prevalence has been reported differently by studies from all over the world. This difference is due to socioeconomic standards, region, cultural practices, and risk behavior (21–23). It was observed in our study that the majority of the WLHIV harbored multiple HR-HPV types in their cervicovaginal secretions. Our findings were similar to other studies, which have reported infection with multiple HPV types in WLHIV (24–29). Infection with multiple HPV types may be due to the persistence of the HPV infection in an immunosuppressed woman with continued sexual exposures to other genotypes, reactivation of latent HPV types, and new infections during periods of immunosuppression. Therefore, women at risk for sexual exposure to HIV are at a higher risk for exposure to genital HPV, and repeated exposures cause infection with multiple genotypes. The oncogenic potential of HPV genotypes-16 and−18 has been studied and reported in the few studies from India that have described HPV genotypes in HIV-positive women. Globally, many studies have reported detecting multiple HPV genotypes in WLHIV as compared with the control group (30).

A high prevalence of other HRHPV (non-16,−18) genotypes, such as HPV-52 and HPV-58, has been reported in WLHIV (31). Some studies have also reported HPV-31,−33, and−56 among the other non-16 and−18 HRHPV genotypes in a similar study cohort (32, 33). Our findings were similar, and the other genotypes seen in our study were HPV-52 (*n* = 17), followed by HPV-74. Two samples were reported as HSIL in our study, one harbored HPV-16 and−52, and the other had a single HR HPV-73. The causal role of these other (non-16,−18) genotypes needs to be investigated.

It was seen in our study subset that CD4 counts were lower in women with HRHPV than those harboring HPV-16. In a cross-sectional study, the prevalence of HRHPV infection was found to be 78.9% in women with a median CD4 count of 125/mm^3^ (34). Since HPV persistence increases with decreasing immune status, HPV-16 was found to be weakly associated with the immune status. A possible explanation for this may be that the immune behavior of each HPV genotype, especially HPV-16, is better adapted to evade the immune system than other types and might play a lesser role in cervical disease in severely immunosuppressed women, whereas genotypes other than−16 might be more aggressive with loss of immunologic control (35).

A study from India on 278 HIV-positive women performed HPV genotyping by the Linear Array assay. One hundred and forty-six (52.5%) women were HPV positive and “carcinogenic” HRHPV types were present in 35.3% (98/278). Half the women had multiple HPV genotypes, while multiple HRHPV genotypes were present in (27.8%) of women with “carcinogenic” HPV (36). Overall, HPV-16 was the commonest genotype in 12% and 47% cervical intraepithelial neoplasia 2 (CIN2) and 50% in CIN3 with a single HRHPV infection. The HRHPV types in descending order of prevalence were HPV-16,−56,−18,−39,−35,−51,−31,−59,−33,−58,−68,−45, and−52. A Northeastern Indian study reported the prevalence of 32.2% of HPV-16,−18 in 93 HIV-positive women. A total of 53% (23/43) of cases with HRHPV were infected with genotypes other than−16,−18 either as single or multiple infections (37).

On comparing HPV genotypes and cervical cytology, we observed that the cytological abnormalities were higher with HPV positivity for HRHPV types (*p* = 0.011) than for HPV-16 alone (*p* = 0.006), and for HPV-16 and/or 18 (*p* < 0.001). The prevalence of abnormal Pap smear in WLHIV has been reported to be five times higher than the general population in which the rate of abnormal Pap smear is 5–6%.

In the present study, 71.36% (*n* = 62) of women had a normal Pap smear cytology. Abnormal cytology was seen in 24.2% of HIV-positive women. It has been reported to be 8–38% in previous studies. A meta-analysis for HPV-positive women (with HIV status unspecified), also showed a pronounced increase in HPV-16 positivity across cytological grades; from normal/ASCUS/ CIN1 (20–28%), through CIN2/HSIL (40/47%), to CIN3/ICC (58/63%) (38). In a worldwide meta-analysis, the proportion of HPV-16 in 19,883 HIV-positive women was 12.6% in women with normal cytology, through 18.3, 24.7, and 32% in those with ASCUS, LSIL, and HSIL, respectively (39).

A study was done at the same institute as this study reported HPV positivity as 7.6, 42.3, and 87.5% in HIV-negative women with normal cytology, LSIL, and HSIL (40). In this study, as expected in a population of HIV-positive women, HPV positivity was substantially higher at 59.7% (37/62) in women with normal cytology, 100% (11/11) in LSIL, and 100% (2/2) in HSIL. While the percentage positivity in this study was similar for HPV-16 in HIV-positive women with normal cytology (19.4%) and ASCUS (25%), but in LSIL, the representation of HPV-16 was much higher (72.7%). A significant difference was seen between cytological abnormalities and PCR hybridization positivity across all groups, with *p*-values ranging from 0.001 to 0.043, which was most pronounced in the HPV-negative group. Since a control group of women without HIV was not included in this study, comparisons were drawn with available data for this group from studies done at the same institute. In a meta-analysis from south Asia, the overall HPV positivity in women with normal cytology was 9–12% and increased to 52 and 76%, respectively, in those with LSIL and HSIL (41).

Quantitative detection of HPV VL for HPV-16 and HPV-18 using real-time PCR was done. HPV VL was normalized to GAPDH for comparability across runs and samples. It was calculated as the ratio between the number of HPV copies, and the number of cells that are present. Normalizing allowed for correction of the VL for the number of target cells/DNA to even out variations due to sampling (42). Since HPV load is a type-dependent marker for cervical cancer, estimating it in case of multiple infections could lead to overestimation. The clinically relevant VL cutoff is not yet decided (43). Deciding on the VL cutoff, which is clinically relevant, would help in the triage of women and identify those who need therapy the most (44). HPV VL measurements for HRHPV especially HPV-16 in cervical specimens have been shown to be a suitable indicator of persistent infection with clinical application (45). In this study, we chose quantitation of HPV-16 and−18 since these are representative of the A9 and A7 clades, having the strongest association with cancer. Real-time PCR targeting the HPV-16 E6 and L1 genes has reported the viral burden and grade of the intraepithelial lesions (46). Studies measuring VL of cervical scrapings at a single time point, normalized for specimen cellularity, did not reveal a consistent association between the HPV VL and the risk of acquiring an epithelial abnormality of the cervix (47). A single measurement of HPV VL cannot be considered a clinically useful biomarker (48). The relative quantitation of the VL may be used as an indicator of disease dynamics (49). High VL or relative increase in copy numbers is associated with an increased risk of epithelial abnormality; however, a single measurement of VL made at an indeterminate point during the natural history of HPV infection does not reliably predict the risk of progression to cervical neoplasia. Also, VL been has seen to wax and vane during serial measurements. Studies have evaluated various tests and assays, which could be used as reliable screening tests (50).

The limitations of our study were the small sample size, which was not large enough to conclude. Studies on a larger cohort would be required to establish the correlation to decide the clinical VL cutoff (51). Prospective studies in HPV-infected women including the determination of VLs would be needed to prove it to be a useful biomarker. In this study, some of the samples positive for HPV DNA by PCR hybridization were not detected by real-time PCR. This occurred even when all precautions were taken for aliquoting and maintaining the appropriate temperature. This may have been due to some deterioration during storage. Another possible explanation for this may be that the gene targets and primers used for the conventional PCR and real-time PCR were different, which may have been a reason for this discrepancy.

Future studies are required to define the optimum frequency for screening intervals in HIV-positive women. In low-resource settings, screening strategies need to be organized based on the available resources. Incorporating self-sampling and POC tests for screening will need to be evaluated and developed into affordable and effective technologies in years to come.

## Conclusion

This study has detected HPV-16 as the commonest genotype in North Indian HIV seropositive women. Genotyping may help in risk stratification of HR types in HPV-associated cervical carcinogenesis. Monitoring of prevalent HPV types will become even more necessary with the widespread use of vaccines. Circulating HPV types may evolve or change as the population is vaccinated, prompting a change in the vaccine-targeted genotypes as well. Epidemiologically, it has implications for the development of newer HPV vaccines. HIV-positive women should be recommended cervical screening by Pap smear where facilities exist. Women should be actively screened by pap smears and HPV-specific molecular tests. Clinically relevant cutoffs of normalized HPV-16, 18 VLs in a high-risk subset of HIV-positive women may be incorporated into cancer screening programs. These in the future would be helpful to guide triage, follow-up, or treatment policies.

## Data Availability Statement

The original contributions presented in the study are included in the article/Supplementary Material, further inquiries can be directed to the corresponding author/s.

## Ethics Statement

The studies involving human participants were reviewed and approved by All India Institute of Medical Sciences, New Delhi. The patients/participants provided their written informed consent to participate in this study.

## Author Contributions

All authors listed have made a substantial, direct and intellectual contribution to the work, and approved it for publication.

## Conflict of Interest

The authors declare that the research was conducted in the absence of any commercial or financial relationships that could be construed as a potential conflict of interest.

## Publisher's Note

All claims expressed in this article are solely those of the authors and do not necessarily represent those of their affiliated organizations, or those of the publisher, the editors and the reviewers. Any product that may be evaluated in this article, or claim that may be made by its manufacturer, is not guaranteed or endorsed by the publisher.
