# Supplementary material for: Prevalence of Human Papillomavirus (HPV) Genotypes in Cervicovaginal Secretions of Human Immunodeficiency Virus (HIV) Positive Indian Women and Correlation With Clinico-Virological Parameters
Source: Front Reprod Health. 2021 Sep 13;3:695254. doi: 10.3389/frph.2021.695254 (PMC9580721; doi:10.3389/frph.2021.695254)
Supplement: Supplementary file 1 [file Data_Sheet_1.docx]

**Table 1: Showing Ct values of HPV-16 Standards**

| **Copies of standard** | **Ct Values** | | **Average Ct Value** |
| --- | --- | --- | --- |
| 10000000 | 17.88 | 18.72 | 18.3 |
| 1000000 | 22.27 | 21.69 | 21.98 |
| 100000 | 26.36 | 26.11 | 26.23 |
| 10000 | 30.77 | 31.04 | 30.90 |
| 1000 | 34.72 | 34.35 | 34.53 |
| 100 | 38.10 | 38.55 | 38.32 |
| 10 | 41.96 | 41.95 | 41.95 |
| NTC | Undetermined | Undetermined | Undetermined |

The tenfold serial dilutions of the constructed plasmid containing the target gene were prepared, from 10^7^ -10^1^ copies/µl, which corresponded to Ct range of 18.3-41.95 on real time PCR. The Ct values of HPV-16 standards measured in duplicate are tabulated (Table 1).

**Table 2: HPV-18 Standards: Ct values**

| **Copies of Standard** | **Ct Values** | | **Average Ct Value** |
| --- | --- | --- | --- |
| 100000000 | 11.71 | 12.25 | 11.98 |
| 10000000 | 17.91 | 18.12 | 18.01 |
| 1000000 | 22.67 | 22.78 | 22.72 |
| 100000 | 26.70 | 26.98 | 26.84 |
| 10000 | 28.92 | 29.46 | 29.19 |
| 1000 | 33.55 | 33.82 | 33.69 |
| 100 | 38.32 | 38.11 | 38.22 |
| 10 | 39.53 | 39.80 | 39.66 |
| NTC | Undetermined | Undetermined | Undetermined |

The linear range of HPV quantification, a tenfold serial dilution of the plasmid containing the target gene (clone) measured in duplicate for HPV-18 is tabulated showing Ct values of HPV-18 standards (Table 2).

**Table 3: Showing ten fold dilution of plasmid containing target gene: GAPDH measured in duplicate; Ct values of GAPDH standards**

| **Copies of standard** | **CT Values** | | **Average CT Value** |
| --- | --- | --- | --- |
| 10000000 | 5.43 | 6.41 | 5.92 |
| 1000000 | 9.97 | 10.33 | 10.15 |
| 100000 | 13.22 | 13.16 | 13.19 |
| 10000 | 16.49 | 16.38 | 16.43 |
| 1000 | 20.31 | 20.11 | 20.21 |
| 100 | 24.33 | 24.19 | 24.26 |
| 10 | 27.59 | 27.08 | 27.33 |
| NTC | Undetermined | Undetermined | Undetermined |

The linear range of GAPDH quantification, a tenfold serial dilution of the plasmid containing the target gene (clone) measured in duplicate for GAPDH was measured and is tabulated showing the Ct values of GAPDH standards (Table 3).
